# Deep mtDNA divergences indicate cryptic species in a fig-pollinating wasp

**DOI:** 10.1186/1471-2148-6-83

**Published:** 2006-10-13

**Authors:** Eleanor R Haine, Joanne Martin, James M Cook

**Affiliations:** 1 Division of Biology, Imperial College London, Silwood Park Campus, Ascot, Berkshire SL5 7PY, UK; 2 Department of Animal and Plant Sciences, University of Sheffield, Western Bank, Sheffield S10 2TN, UK

## Abstract

**Background:**

Figs and fig-pollinating wasps are obligate mutualists that have coevolved for *ca *90 million years. They have radiated together, but do not show strict cospeciation. In particular, it is now clear that many fig species host two wasp species, so there is more wasp speciation than fig speciation. However, little is known about how fig wasps speciate.

**Results:**

We studied variation in 71 fig-pollinating wasps from across the large geographic range of *Ficus rubiginosa *in Australia. All wasps sampled belong to one morphological species (*Pleistodontes imperialis*), but we found four deep mtDNA clades that differed from each other by 9–17% nucleotides. As these genetic distances exceed those normally found within species and overlap those (10–26%) found between morphologically distinct *Pleistodontes *species, they strongly suggest cryptic fig wasp species. mtDNA clade diversity declines from all four present in Northern Queensland to just one in Sydney, near the southern range limit. However, at most sites multiple clades coexist and can be found in the same tree or even the same fig fruit and there is no evidence for parallel sub-division of the host fig species. Both mtDNA data and sequences from two nuclear genes support the monophyly of the "*P. imperialis *complex" relative to other *Pleistodontes *species, suggesting that fig wasp divergence has occurred without any host plant shift. Wasps in clade 3 were infected by a single strain (W1) of *Wolbachia *bacteria, while those in other clades carried a double infection (W2+W3) of two other strains.

**Conclusion:**

Our study indicates that cryptic fig-pollinating wasp species have developed on a single host plant species, without the involvement of host plant shifts, or parallel host plant divergence. Despite extensive evidence for coevolution between figs and fig wasps, wasp speciation may not always be linked strongly with fig speciation.

## Background

Hosts and their symbionts often have major effects on each other's evolution. Indeed, many symbioses show coevolution of key traits, such as parasite virulence and host resistance and, in some cases, may also manifest cospeciation. A classic example of a coevolved mutualism is provided by the obligate relationship between fig trees (*Ficus *species) and fig-pollinating wasps (Hymenoptera:Agaonidae). Female wasps enter receptive fig syconia (inflorescences) via a narrow opening called the ostiole, pollinate the flowers and lay their eggs inside developing fig ovules. The fig wasp offspring develop and then mate inside the syconium, before the next generation of females disperses carrying pollen to other, receptive syconia. Agaonid wasps are the only vectors for fig pollen, and fig syconia the only breeding ground for the wasps, making this an obligate association for both partners. As expected, there are clear examples of coadaptation between corresponding fig and pollinator traits (e.g. [1-5]).

There are over 750 species of figs worldwide [6] and most have only one recorded pollinator species [4,7]. Similarly, most wasp species have only one recorded fig host, leading to the famous 1:1 rule of reciprocal partner specificity. Comparisons of the phylogenies of figs and fig wasps support a long history of co-radiation [8] and also show that cospeciation has played a significant role [9]. However, they do not support strict cospeciation, as is found in some symbioses, such as that between aphids and *Buchnera *bacteria [10]. In addition, recent work has highlighted biases against detecting cases that break the 1:1 specificity rule [11] and revealed many cases where a single fig species hosts two (or occasionally more) pollinator species [12-15]. It now appears that a substantial minority of fig species have two or more co-pollinators [11,15]. In some cases, they are largely allopatric, but in many they coexist in sympatry [11,12,15].

In some cases, co-pollinators have been identified during taxonomic revisions following extended field sampling [13], while in others they were initially identified via surprising patterns of genetic variation within what was thought to be a single wasp species [14]. Regardless of how they are identified, co-pollinators have important consequences. First, they change our view of host specificity and make coevolutionary dynamics more complex [11,14,15]. Second, their coexistence in the same specialised niche poses a problem for ecological competition theory [16]. Third, fig wasps provide a model system for sex ratio studies and past work has involved accidental pooling of members of two species [14,17].

The occurrence of co-pollinators raises the question of how they evolved. They may be sister species that speciated on the current host plant, or less closely related because one underwent a host-shift from another fig species. While host-shifting has been important in the radiation of many herbivorous insect taxa (e.g. [18-20]), it is less clear how a fig-pollinating wasp might speciate without a host-shift or host plant speciation event. One potentially important agent is *Wolbachia*, an alpha-proteobacterium that often causes reproductive incompatibilities between infected and uninfected hosts, or between populations with different mutually incompatible infections. In theory, these can facilitate – or even cause – host speciation [21-25]. Fig-pollinating wasps have the highest known incidence of *Wolbachia *infection for any insect taxon with *ca*. 70% of Australian and Panamanian species harbouring infections [26,27]. Interestingly, we have previously detected variation in infection status in the fig wasp *Pleistodontes imperialis *during a wide survey of fig wasp species [27].

Most previous studies of fig/pollinator specificity have been either general literature surveys [7,28,29], or detailed studies of a few species at one or a few sites (e.g. [13,14]). These, respectively, revealed geographic variation in pollinator species and local coexistence of alternative pollinators. Here, we combined these two approaches by studying one fig species (*Ficus rubiginosa*) with a large geographic range, and collecting many fig-pollinating wasps (*Pleistodontes imperialis*) from several sites across that range. We used sequences from four different genetic markers – one mitochondrial (cytochrome b) and two nuclear (28S and wingless) wasp genes and one *Wolbachia *(wsp) gene to explore the genetic variation in *P. imperialis *across the large geographic range of its host plant.

**Figure 1:**
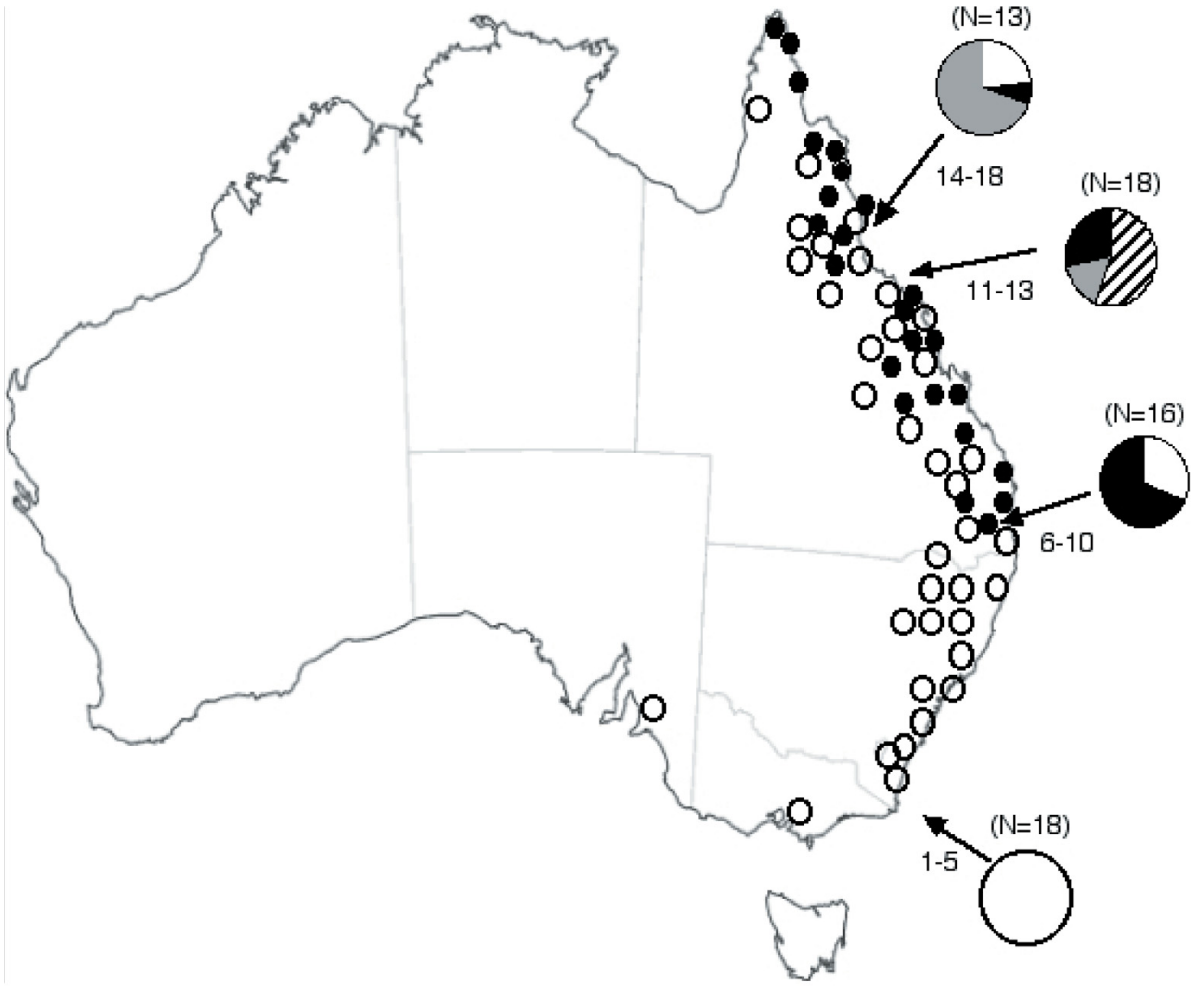
**Distribution and frequencies of the four *P. imperialis *mtDNA clades**. Distribution of clades 1 (white fill), 2 (hatch), 3 (gray) and 4 (black) in four regions across the range of *F. rubiginosa*. Sample sizes are given in parentheses above pie-charts. Numbers next to arrows refer to sampling sites listed in Table 1. Open and closed circles on the map reveal the distributions of *F. rubiginosa *f. rubiginosa and *F. rubiginosa *f. glabrescens, respectively. QLD = Queensland, NSW = New South Wales, VIC = Victoria, SA = South Australia.

*Ficus rubiginosa *occurs naturally along the Eastern Coast of Australia (roughly 2500 km North-South and up to 200 km inland) and is found in diverse habitats, including rainforest, granite outcrops and rocky coastal areas (Fig. 1; [30]). It is also commonly planted in parks and there are introduced populations in other parts of Australia (e.g. Adelaide and Melbourne), as well as in New Zealand [31], Hawaii [32], California and Mediterranean Europe (JMC, pers. obs.). In a recent taxonomic revision of species in *Ficus *section Malvanthera, *F. rubiginosa *was considered to have two forms with one difference: form *rubiginosa *has leaves that are variously hairy, while form *glabrescens *[30] lacks hairs. However, individual leaves of form *rubiginosa *may also lack hairs. Form *rubiginosa *has a natural distribution from Cape York down the East Coast of Australia to Southern New South Wales (NSW), while form *glabrescens *has the same northern distribution, but does not extend south into NSW (Fig. 1). Only one pollinator wasp species, *Pleistodontes imperialis*, has been recorded, despite extensive sampling and a recent taxonomic revision of the wasp genus [13], which led to the description of four new *Pleistodontes *species from other fig species. Most *P. imperialis *females are black, but a yellow form is found around Townsville in N. Queensland. Morphological analysis revealed no clear differences, except for colour, and they are considered to be the same species [13]. *Pleistodontes imperialis *has not been recorded from any other fig species [13].

## Results

### Phylogenetic patterns of Cytochrome B variation

We analysed 71 *P. imperialis *wasps from 26 different *F. rubiginosa *trees, representing 18 sites in Australia and one introduced population in the USA (Table 1, Fig. 1). We found 44 unique *cytb *haplotypes, 444 bp in length, of which 139 (31.1%) nucleotide sites were polymorphic and 107 (24.1%) parsimony-informative.

**Table 1:** Summary of *P. imperialis *samples and their genetic characteristics.

**Location**	**Site**	**Tree ID**	**N**	***Cytb *clade**	***Wolbachia *strain**	***28S *group**	**Wingless group**	**Colour**
Adelaide	1	NI2	3	1	W2W3	I	wgB	Black
Melbourne	2	jmc01–52	3	1	W2W3	-	-	Black
	2	jmc01–53	3	1	W2W3	-	-	Black
	3	eh02–10	1	1	W2W3	I	wgB	Black
	3	eh02–29	2	1	W2W3	I	wgB	Black
	3	eh02–40	2	1	W2W3	-	-	Black
Sydney	4	eh02–20	1	1	W2W3	I	-	Black
	5	NI1	3	1	W2W3	I	wgB	Black
Brisbane	6	jmc03–55	3	4	W2W3	II	wgB	Black
	6	jmc03–56	1	4	W2W3	II	wgB	Black
	7	pcam11	3	1	W2W3	-	-	Black
	8	jmc03–61	3	4	W2W3	II	wgB	Black
	9	pcam12 1/3	2	4	W2W3	-	-	Black
	9	pcam12 2	1	1	W2W3	I	wgB	Black
	10	NI5 2	1	1	W2W3	I	wgB	Black
	10	NI5 1/3	2	4	W2W3	II	wgB	Black
Townsville	11	jmc03–35	3	2	W2W3	I	wgB	Yellow
	11	eh02–33	5	4	W2W3	II	wgB	Black
	12	jmc01–41	3	3	W1	I	wgA	Black
	13	eh02–38	4	2	W2W3	I	wgB	Yellow
	13	eh02–41 2/3	2	2	W2W3	I	wgB	Yellow
	13	eh02–41 1	1	2	W2	-	-	Yellow
Atherton	14	jmc01–28	3	1	W2W3	I	wgB	Black
Tablelands	15	jmc03–28 1/3	2	3	W1	I	wgA	Black
	15	jmc03–28 2	1	4	W2W3	II	wgB	Black
	16	jmc03–25	3	3	W1	I	wgA	Black
	17	jmc03–22	3	3	W1	I	wgA	Black
	18	eh02–24	1	3	W1	I	wgA	Black
San Diego		NI6	3	1	W2W3	-	-	Black
		NI7	3	1	W2W3	-	-	Black

N = no. of wasps typed; - = not determined for this sample; *cytb *clade indicates into which of 4 major clades the *cytb *sequences fell, see main text for further details; individuals were infected by three different *Wolbachia *strains, W1, W2 and W3; *28S *sequences and wingless sequences each fell into two groups, indicated here by I and II, and wgA and wgB, respectively.

**Figure 2:**
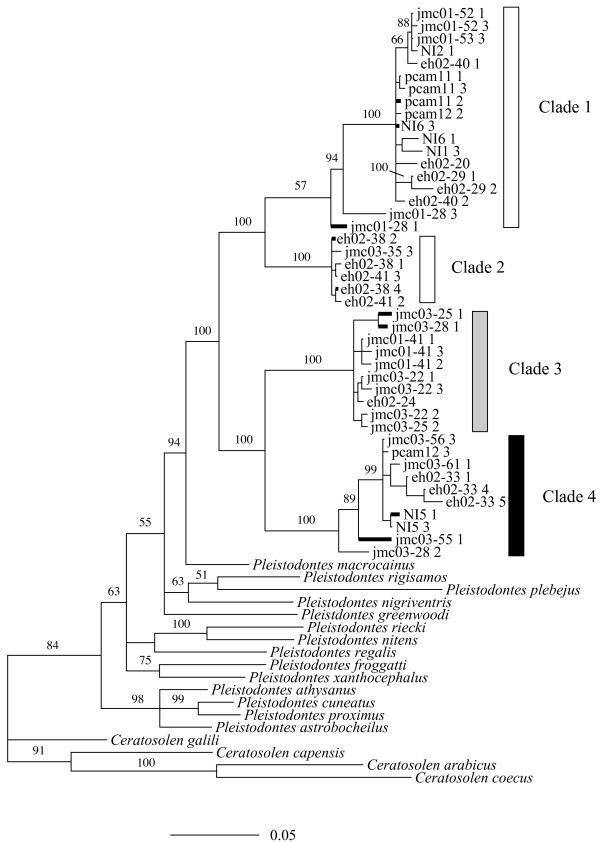
**Cytochrome b**. Consensus Bayesian topology of a 444 bp region of the cytochrome b gene for 45 *P. imperialis *individuals, 14 other *Pleistodontes *species [33] and 4 outgroup *Ceratosolen *species [34]. Posterior node probabilities are indicated above each node. Thick branches represent >1 identical haplotypes.

Forty-four *P. imperialis *sequences were used in phylogenetic analyses, together with sequences from 14 other *Pleistodontes *species [33] and four *Ceratosolen *fig-pollinating wasp species [34] as outgroups. The MP (not shown) and Bayesian phylogenies (Fig. 2) had very similar topologies, both supporting the monophyly of *P. imperialis*, but dividing the species into four deep mitochondrial clades. The (GTR) pairwise genetic distances between taxa fell into two distinct groups: 1) distances within each of the four *P. imperialis *clades (0–7%); and 2) distances between *P. imperialis *clades (9–17%) and between morphologically distinguishable *Pleistodontes *species (10–26%). Consequently, the large genetic distances between the four *P. imperialis *clades are similar to those between morphologically distinct *Pleistodontes *and indicate cryptic species. There was one divergent *P. imperialis *sample, from which three wasps grouped only weakly with the rest of clade 1 (Figure 2). Based upon the commonly used mitochondrial DNA (mtDNA) clock rate of 2.3% pairwise divergence/Myr [35], clades 1 and 2 diverged from clades 3 and 4 at least 7.1 million years ago (MYA), clades 3 and 4 split 5.6 MYA and clades 1 and 2 split 4.4 MYA

### Geographic distribution of Cytochrome B clades

The four *cytb *clades show different geographic distributions (Fig. 1). Clade 1 was found throughout the natural range (Queensland (except Townsville) and New South Wales (NSW)) of *P. imperialis *and was also the only clade found outside the native range (in S. Australia, Victoria and USA). Clade 2 was found only in the Townsville area of Northern Queensland and contained all the yellow wasps that are also restricted to this area. Clade 3 wasps were found only in N. Queensland, while clade 4 was found in N. and S. Queensland, but not in NSW. Overall, clade diversity appears to decrease from North to South (Fig. 1).

There is not, however, simple geographic replacement of clades, as most sites in Queensland have two or more clades present. For example, wasps collected from just five trees near Townsville represent three of the four clades (Table 1). In most cases, all individuals from one fig tree belonged to the same clade. However, in three cases individuals from the same tree belonged to two different clades (Table 1). Given that only 3–5 wasps were sampled per fig tree, this suggests the frequent occurrence of wasps from two or more clades in a single crop of fig fruits.

### Wolbachia infections

All 71 wasps harboured *Wolbachia*, but some carried one strain and others two. We obtained *wsp *sequences from 27 insects, revealing 6 with single and 21 with double infections (Table 1). The *wsp *sequences all belong to the *Wolbachia *A-clade, and revealed three strains (W1, W2 and W3). W1 and W2 strains differed by a single synonymous A/G substitution at position 268, while there was 8.5% nucleotide divergence, including a 3 bp and a 21 bp indel between W2 and W3. There was a highly consistent pattern between *Wolbachia *infection status and *cytb *clades. Wasps in clade 3 had a single infection with W1, while wasps from the other three clades all had both the W2 and W3 infections. The single exception was one clade 2 wasp that harboured only strain W2.

### Nuclear sequence variation

**Figure 3:**
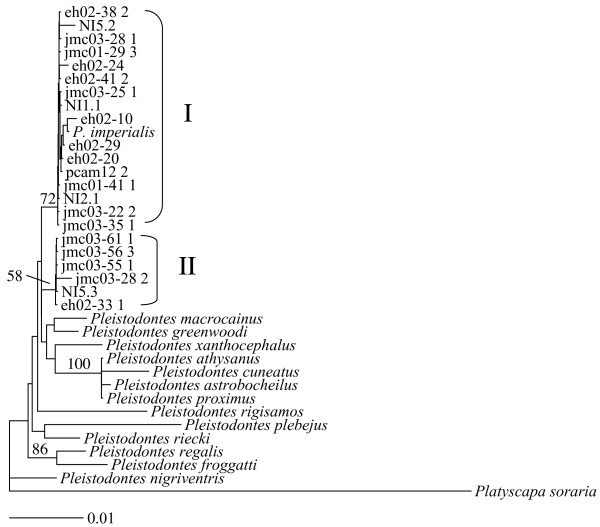
**28S rRNA**. Neighbour-joining phylogram of a 1,060 bp region of the 28S rRNA gene for 23 *P. imperialis *individuals, 14 other *Pleistodontes *species and an outgroup taxon, *Platyscapa soraria*. Percentage bootstrap support (1000 replicates) is indicated above branches. Unlabelled branches had bootstrap support of less than 50%.

We sequenced nuclear *28S *(1033 bp) and *wg *(433 bp) DNA fragments for 22 wasps representing the four *cytb *clades (Table 1). The *28S *data revealed two weakly supported clades (28S.I and 28S.II) that differed from each other by 4–8 nucleotide substitutions, while there was a maximum of 2 base differences between wasps within the two clades (Fig. 3). 28S.II contains individuals from cytb clade 4 only, while 28S.I individuals fall into *cytb *clades 1–3 (Table 2). *Wg *also divided the wasps into two clades (wgA and wgB) and these differed from each other by a single nucleotide substitution at a synonymous site (position 175) (0.23% nucleotide divergence). Group wgA contained individuals from cytochrome b clade 3 only, while wgB individuals belonged to clades 1, 2 and 4 (Table 2). To place this in context, we also sequenced *wg *from eight outgroup taxa: four other *Pleistodontes *species and four *Ceratosolen *species. Nucleotide divergence ranged from 1.16% to 3.23% between morphologically distinguishable *Pleistodontes *species and up to 16% between genera. While there is no clear association between *28S *variation and *Wolbachia *infection, there is an association of *Wolbachia *with genetic divergence of *wg *between clade 3 (W1 infection; wgA) and clades 1, 2 and 4 (W2 and W3 infections; wgB). The overall correspondence of different markers is summarised in Table 2.

**Table 2:** Correspondence between groupings of wasps using three insect genes, *Wolbachia *infection status and wasp colour.

***Cytb *Clade**	**Geographic distribution**	**Wingless group**	***28S *group**	***Wolbachia *strain**	**Wasp Colour**	**N^†^**
1	Entire Range	WgB	I	W2W3	Black	14
2	Townsville area	WgB	I	W2W3	Yellow*	4
3	Atherton Tablelands, Townsville area	WgA*	I	W1*	Black	5
4	All Queensland	WgB	II*	W2W3	Black	7

Asterisks indicate other marker character states that support isolation and integrity of mtDNA *cytb *clades. ^†^Number of independent samples (i.e. syconia) from which individuals were derived.

## Discussion

### Deep mtDNA divergences within "*P. imperialis*"

We have demonstrated that *P. imperialis *wasps fall into four major mtDNA clades. These differ from each other by 9–17% nucleotides and are supported strongly by both MP and Bayesian phylogenetic analyses (Fig. 2). These deep divergences are very similar to those (10–26%) found between morphologically distinct members of the same genus [33]. The mtDNA genetic distances between our clades are also slightly higher than those reported between cryptic species of *Pegoscapus *fig-pollinating wasps in Panama [14]. These deep divergences suggest strongly the existence of four cryptic species within the morphologically defined *P. imperialis*.

We also showed that all wasps harboured *Wolbachia *bacteria and that clade 3 wasps had a different infection to all other wasps. *Wolbachia *can influence both the diversity and evolution of mtDNA and can maintain mtDNA divergences within or between populations of a species [36]. The best-studied example is *Drosophila simulans *which has three distinct haplotypes [37] and is infected by at least five strains of *Wolbachia *[38-43]. However, in this and other cases where populations are polymorphic for *Wolbachia *infection (e.g. the gall wasp *Biorhiza pallida *[44]) the genetic distances between different haplotypes (clades) are very much lower than we report here. Nevertheless, given the very high incidence of *Wolbachia *infections in fig-pollinating wasps [26,27], these endosymbionts have probably played a role in host mtDNA evolution and reduce confidence in the applicability of a general mtDNA clock.

### Evidence against a role for host shifts

Two wasp species may co-occur on a single fig species if one of them has shifted from another host fig. Indeed, some mismatches between figs and wasps at deep phylogenetic levels suggest that host shifts have occurred at times during their coevolutionary history [9,15]. In addition, there are cases of extant co-pollinators that are not closely related species, suggesting more recent host shifts (e.g. [14,34]. However, despite high genetic distances between the four cytb clades, all three wasp genes sequenced support the monophyly of the *P. imperialis *complex relative to other *Pleistodontes *species. Consequently, it seems unlikely that any of our four clades has shifted to *F. rubiginosa *from another *Ficus *species. In addition, we have not recorded *P. imperialis *from another fig species, nor detected mitochondrial haplotype groups shared by *Pleistodontes *wasps from different fig species ([33] and further unpublished data).

### Evidence against parallel divergences of *F. rubiginosa *and *P. imperialis*

Alternatively, the *F. rubiginosa/P. imperialis *species pair could be in the process of cospeciation, with the partner species diverging together. However, our existing data do not support this notion and it is also difficult to envisage how the fig species might split simultaneously into four genetic units. We found that in most areas, and even some individual trees, two or more wasp clades were present (Table 1), despite limited within-site sampling. Furthermore, as part of a long-term survey in Townsville, we have found that most *F. rubiginosa *syconia are entered by more than one foundress and that yellow (clade 2) and black wasps (clades 3 or 4 at this site) co-enter about 20% of syconia (53/198 figs from 10 different trees over 2 years). These results suggest that there is no simple segregation of wasp clades between different host trees and that they co-occur regularly in the same trees and even fruits, as reported in Panama for cryptic species of *Pegoscapus *fig-pollinating wasps [14,17].

One possibility is that the different wasp clades predominate in different habitats and that the fig species is diverging into habitat-specific races. For example, in West Africa *Ficus ottonifolia *occurs in a mosaic of forest and open habitats and has two pollinators. These co-occur locally, but one predominates in open habitat patches and the other in forest patches [12,45]. *F. rubiginosa *is also found in both forest and open habitats and further sampling might reveal a similar pattern. However, we believe that this is unlikely as, for logistical reasons, almost all of our samples are from open habitats.

A further possibility is that the fig is diverging into two or more races that are not habitat-specific. Indeed, Dixon et al. (2001) recognised two forms of *F. rubiginosa *(see background). Most of our sampling was performed before the description of these two forms, so we cannot yet compare pollinators between the different forms. However, most of our samples came from form *rubiginosa*, arguing against a simple split by form. In addition, the only taxonomic character that separates the two forms is the presence of hairs on the leaves, which may well be a simple polymorphism that has no connection with the pollinators. Finally, even if there is segregation by fig form, this can only provide a partial answer since there are four wasp clades, but only two fig forms. Clearly, genetic studies of the figs are needed to test ideas further. However, we note that recent genetic studies of wasps have revealed cryptic species [14], but genetic studies of the corresponding figs have not [8,15].

### Wasp divergence without direct involvement of host plant

We argue above that there is no good evidence for either parallel divergence of *F. rubiginosa *and *P. imperialis*, or for recent host shifts by wasps. Fig wasp divergence might instead occur following the development of spatial or temporal barriers within a single wasp species. For example, temporary geographic isolation of wasp (and fig) populations could occur for periods of time that allow the evolution of reproductive isolation in the wasps, but not the figs. Selection and/or genetic drift may be involved and the approximately 100 times faster generation time of the insects may facilitate their population divergences [11]. Many scenarios are possible, but we suggest that the role of *Wolbachia *deserves further study, since we have detected infection differences and the acquisition of different *Wolbachia *infections in isolated populations can facilitate or even cause speciation [23-25].

### Correspondence of other markers with mtDNA clades

There is very little variation in the slower-evolving nuclear genes studied. However, *28S *sequences split the wasps into two clades that correspond to *cytb *clades 1–3 and clade 4, while *wg *also splits them into two groups, but corresponding to *cytb *clade 3 and clades 1, 2 and 4. *Cytb *clade 3 stands out further by differing in its *Wolbachia *infection status. Consequently, three markers support isolation of clade 3 from the others, and clade 4 differs from all others on the basis of variation in *28S*. Clades 1 and 2 differ strongly in mtDNA, but not in the other genes studied. However, clade 2 contains only (and all) the yellow wasps sampled, while all other wasps are black and this effectively provides a nuclear marker supporting isolation of clade 2.

Further resolution of gene flow in the "*P. imperialis *complex" now requires data from from substantial numbers of wasps representing the different clades. Given the limited variability of the nuclear sequences studied here, a population genetic approach, using microsatellites, may be most appropriate.

## Conclusion

Our study reveals deep mtDNA divergences within *P. imperialis *and indicates the presence of cryptic species. The data further suggest that these fig-pollinating wasps have diverged without a role for host plant shifts or corresponding fig speciation. This adds to the growing body of evidence that figs and their pollinators have not radiated simply by strict cospeciation. Recent studies of figs in sections *Americana *and *Pharmacosycea *have revealed that some wasp species are regularly associated with two fig species and that host shifts may be common. There is also support for past hybridisation of at least one pair of these fig species linked by a common wasp species. Consequently, Machado et al. [15] suggested a revised coevolutionary model involving groups of genetically well-defined wasp species coevolving with groups of genetically less well-defined (frequently hybridizing) groups of figs. Current data on *Malvanthera *figs and their *Pleistodontes *pollinators support the notion of genetically well-defined wasp species, but we have not found regular sharing of pollinator species between distinct fig species, despite frequent coexistence of multiple wasps on one fig species. In addition, the few obviously hybrid trees we know are cultivated rather than wild. However, we now need genetic studies of potentially hybridising fig species, along with continuing surveys of pollinator specificity, to assess whether this new model applies widely to figs and fig wasps. In summary, there are at least two ways – host shifting and independent wasp speciation – to evolve co-pollinators and break the 1:1 rule of host specificity.

## Methods

### Field sampling

Between 1998 and 2003 ripe syconia were collected from *F. rubiginosa *trees growing at many disparate locations throughout its natural range. Wasps were allowed to emerge naturally from their syconia and were then stored in 95% ethanol at -20C. A single collection tube comprised wasps from syconia from the same tree on the same day. We later conducted genetic analyses on 1–5 female wasps from each sample tube.

### Molecular methods

The head from each insect was removed and retained as a voucher specimen, and DNA was extracted from the remaining body parts using a simple Chelex extraction procedure [46]. We amplified a 444 bp fragment of mitochondrial cytochrome b (*cytb*) for all wasps using the primers CB1 and CB2 [47]. For selected individuals (see results), we also amplified a region of the nuclear 28S rRNA gene (*28S*), using primers D1F and D3R [33,48,49] as well as the wingless (*wg*) gene, using primers LepWG1 and LepWG2 [50]. All wasps were also screened for *Wolbachia *infection by PCR, employing the primers wsp81F and wsp 691R, which amplify part of the *Wolbachia *surface protein gene (*wsp*) [51].

Amplification of *cytb *was performed using a GeneAmp 2400 machine (Perkin-Elmer Cetus) with 3 min at 95°C, followed by 35 cycles of 30 s at 95°C, 1 min at 45°C, 1 min 30 s at 72°C, and a final elongation step of 7 min at 72°C. We increased the annealing temperature for the other gene fragments as follows: 55°C for *wsp*, 50°C for *28S *and 58°C for *wg*. The sizes of PCR products were 444 bp (cytb), 564–588 bp (*wsp*), ~1,060 bp (*28S*), and 433 bp (*wg*). Ten microlitres of each PCR product was electrophoresed through a 1% agarose gel to determine amplicon size, and the gel band was excised for purification using a GFX DNA Purification Kit (Amersham Pharmacia Biotech Inc). We then sequenced fig wasp genes (*cytb*, *28S *and *wg*) directly using the same primers employed in PCR.

For *wsp*, PCR products were first sequenced directly and, if direct sequencing failed three times (or repeatedly generated sequences with multiple peaks), the PCR product was cloned and 6–10 different clones were sequenced to test for the presence of multiple *Wolbachia *strains. We ligated each PCR product into a T-tailed vector (pGEM-T Easy Vector system, Promega Ltd.) and transformed into *E. coli *JM109. Positive colonies were selected and plasmid DNA was purified using a GFX Micro Plasmid Prep Kit (Amersham Pharmacia Biotech Inc) and the *wsp *inserts were sequenced using M13 vector primers. In all cases, we sequenced using the ABI PRISM BigDye Terminator Cycle Sequencing Kit (Perkin Elmer Inc.) and an ABI PRISM 3700 DNA Analyzer (Perkin Elmer Inc). All isolates were sequenced fully in both directions, and sequences have been deposited in GenBank: *cytb *[GenBank: AY567594–AY567638], *28S *[GenBank: AY567639–AY567660] and *wg *[GenBank: DQ539361–DQ539391].

### Sequence alignment and phylogenetic analysis

Sequences were edited and aligned using Sequencher™ (Gene Codes Corporation), and final adjustments to *28S *and *wsp *alignments were made by eye, following previous alignments (*wsp*: Shoemaker et al. 2002; *28S*: Lopez-Vaamonde et al. 2001).

*Cytb *phylogenies were estimated using Maximum Parsimony (MP) in PAUP* version 4.0b10 [52], and Bayesian methods in MRBAYES version 3.0 [53]. For MP analyses, we conducted an initial heuristic search with 10,000 random additions and TBR branch swapping, holding one tree per replicate. Trees generated by the initial search were then used as starting trees for a second heuristic search, in which multiple trees were saved. We assessed clade support using 1000 bootstrap replicates. For Bayesian analyses, the most appropriate model of nucleotide substitution was determined, using MrModeltest v2.2 [54], to be the general time reversible model (nst = 6). The analyses were run for 10^6 ^generations, with one tree retained every 100 generations. Likelihood stationarity occurred after 2.5 × 10^5 ^generations, and this "burn-in" period was excluded before creating a 50% majority-rule consensus tree in PAUP*.

## Authors' contributions

ERH and JMC designed the study and conducted field sampling. ERH carried out most of the molecular genetic studies and all data analyses. JM generated sequence data for the wingless gene. ERH led the writing of the original submission and JMC led the revision following referees' comments. All authors read and approved the final manuscript.
